# Pathophysiology of Physical Inactivity-Dependent Insulin Resistance: A Theoretical Mechanistic Review Emphasizing Clinical Evidence

**DOI:** 10.1155/2021/7796727

**Published:** 2021-10-07

**Authors:** Habib Yaribeygi, Mina Maleki, Thozhukat Sathyapalan, Tannaz Jamialahmadi, Amirhossein Sahebkar

**Affiliations:** ^1^Research Center of Physiology, Semnan University of Medical Sciences, Semnan, Iran; ^2^Urology and Nephrology Research Center, Shahid Beheshti University of Medical Sciences, Tehran, Iran; ^3^Academic Diabetes, Endocrinology and Metabolism, Hull York Medical School, University of Hull, UK; ^4^Department of Food Science and Technology, Quchan Branch, Islamic Azad University, Quchan, Iran; ^5^Department of Nutrition, Faculty of Medicine, Mashhad University of Medical Sciences, Mashhad, Iran; ^6^Biotechnology Research Center, Pharmaceutical Technology Institute, Mashhad University of Medical Sciences, Mashhad, Iran; ^7^Applied Biomedical Research Center, Mashhad University of Medical Sciences, Mashhad, Iran; ^8^School of Pharmacy, Mashhad University of Medical Sciences, Mashhad, Iran

## Abstract

The modern lifestyle has a negative impact on health. It is usually accompanied by increased stress levels and lower physical activity, which interferes with body homeostasis. Diabetes mellitus is a relatively common metabolic disorder with increasing prevalence globally, associated with various risk factors, including lower physical activity and a sedentary lifestyle. It has been shown that sedentary behavior increases the risk of insulin resistance, but the intermediate molecular mechanisms are not fully understood. In this mechanistic review, we explore the possible interactions between physical inactivity and insulin resistance to help better understand the pathophysiology of physical inactivity-dependent insulin resistance and finding novel interventions against these deleterious pathways.

## 1. Introduction

The global prevalence of diabetes mellitus (DM) is growing rapidly [1]. This metabolic disorder is responsible for more than a dozen debilitating complications that negatively affect the quality of life and detrimentally impact various crucial organs such as the kidneys, nervous system, and cardiovascular system [2, 3]. The exact pathophysiology of DM is unclear, but the role of insulin resistance, especially in type 2 DM, is well confirmed [4]. Some lifestyle-dependent factors facilitate the development of insulin resistance, and its incidence is rapidly growing even among young adults [4–6]. Insulin resistance was previously considered an aging problem. But substantial changes in modern lifestyle towards lower physical activity have also increased the prevalence of DM in young adults [6, 7]. Furthermore, there is a positive relationship between long-term physical inactivity and insulin resistance [4, 8]. But the exact linking pathophysiologic pathways are not well understood. The current study is aimed at introducing both confirmed and potential molecular mechanisms by which physical inactivity induces insulin resistance.

## 2. Insulin Signaling Pathway and Insulin Resistance

**Figure 1:**
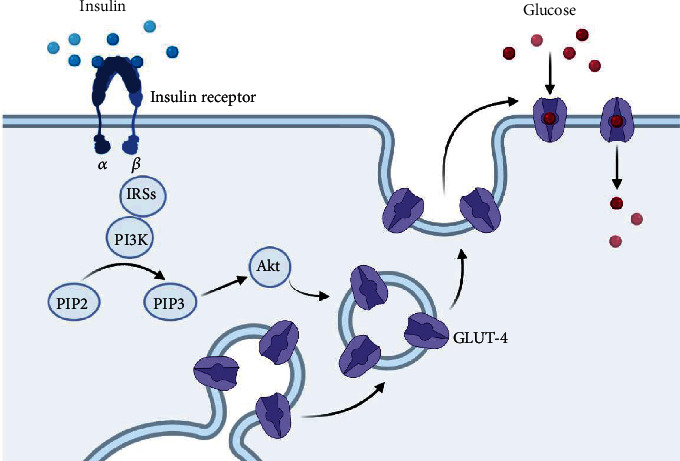
Simple schematic pic of insulin signal transduction (IRSs = insulin receptor substrates; PI3K = phosphoinositide 3-kinase; PIP2 = phosphatidylinositol 4,5-bisphosphate; PIP3 = phosphatidylinositol 3,4,5-trisphosphate; Akt = protein kinase B; Glut-4 = glucose transporter type 4).

Insulin is a peptide hormone released by islets of pancreatic beta cells [5]. This hormone has significant effects on metabolic pathways, and thereby, it is critical for normal homeostasis of the body metabolism [5]. Insulin acts via complicated sequential steps known as insulin signal transduction (IST) which starts by binding insulin to the *α* chain of insulin receptor (IR), which is a transmembrane tyrosine kinase composed of two chains as *α* and *β* [9]. This process stimulates autophosphorylation in the *β* chain, which in turn recruits different adaptor proteins such as IRSs (insulin receptor substrates), Shc protein (SHC-transforming), and APS protein (adapter protein with a PH and SH2 domain) [10, 11]. These events provide a suitable binding site for IRS-1 (insulin receptor substrate-1) and activate it, which links to PI3K (phosphoinositide 3-kinase) and catalyzes the conversion of PIP_2_ (phosphatidylinositol 4,5-bisphosphate) to PIP_3_ (phosphatidylinositol 3,4,5-trisphosphate) [11, 12]. In addition, PIP_3_ is itself a potent activator for PKB (protein kinase B, also known as Akt), which facilitates glucose entering into the cells by localization of GLUT-4 (glucose transporter type 4) [12, 13] (Figure 1). Any defect in these pathways may lead to impaired insulin-dependent glucose entering the cells, known as insulin resistance in adipocytes and skeletal muscles [4].

## 3. Pathophysiologic Links between Physical Inactivity and Insulin Resistance

**Figure 2:**
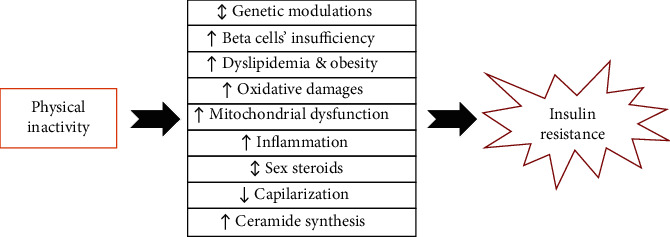
Possible links between physical inactivity and insulin resistance.

There is considerable evidence to emphasize the relationships between physical inactivity and insulin resistance [14]. However, the exact pathophysiological links are not clear so yet. Thus, in the following paragraphs, we will discuss the possible relationships based mainly on clinical evidence (Figure 2).

### 3.1. Genes and Proteins Involved in Glucose Homeostasis

As described before, IST is included of a variety of proteins and enzymes which all work together to facilitate glucose entry into the insulin-dependent cells [9]. Any defect in these harmonic processes will potentially reduce insulin sensitivity [9]. People with insulin resistance have a point mutation or dysfunction in their IST elements [15–17]. Mutation in a single gene or dysfunction of an enzyme such as Akt, PI3K, or IRs could potentially result in impaired IST and contributes to the development of insulin resistance and DM [16]. Hence, proper functioning of this pathway plays an important role in insulin sensitivity and, in turn, glucose homeostasis [17].

Physical inactivity negatively affects the expression, translocation, and function of genes/proteins involved in glucose homeostasis [18–20]. On the other hand, aerobic exercise is a potent stimulus for these genes [4, 21]. For example, Glut-4 is the main route of glucose entry for insulin-dependent cells and has a crucial role in IST [22]. As a result, any disturbance in its expression or function could disrupt insulin signaling and results in insulin resistance [22–24]. Vukovich and colleagues in 1996 showed that even six days of physical inactivity reduces insulin action, which was analyzed via the hyperinsulinemic-euglycemic clamp method, through lowering Glut-4 levels in muscles of endurance-trained runners [25]. Also, Alibegovic and coworkers in 2010 demonstrated that physical inactivity-dependent insulin resistance is related to lower levels of Glut-4 expression in skeletal muscles of young men [19]. They measured insulin sensitivity by the hyperinsulinemic-euglycemic clamp method. They found that 9 days of complete bed rest significantly impacts genes involved in insulin signaling, such as Glut-4, HK2 (hexokinase 2), RRAD (Ras-related glycolysis inhibitor and calcium channel regulator), and TXNIPy, which decreases insulin sensitivity in skeletal muscles [19]. Chibalin et al. in 2000 provided other experimental evidence demonstrating that physical activity increases IRS-1, IRS-2, Akt, PI3 kinase, and Glut-4 expression in rats [26]. Moreover, Biensø et al. in 2014 found the same results by assessing the possible role of 7 days of bed rest intervention on the expression of genes involved in glucose homeostasis [18]. They observed that physical inactivity downregulates Glut-4, HK2, GS (glycogen synthase), and Akt proteins and reduces insulin sensitivity which was examined by the euglycemic-hyperinsulinemic clamp method in skeletal muscles of young, healthy men [18].

Glynn et al. reported in 2008 that decreased levels of physical activity are associated with higher IRS-1 serine phosphorylation and lesser insulin sensitivity, while chronic exercise (running wheels for 9 weeks) reverses these changes in skeletal muscles of rats [27]. Bunprajun et al. in 2013 have shown that physical activity prevents insulin resistance by promoting the Glut-4 expression/translocation in middle-aged volunteers [28]. They demonstrated that active individuals have higher Glut-1 and Glut-4 mRNA expression and higher Glut-4 protein levels in skeletal muscles than sedentary individuals [28]. This evidence suggests that exercise has a pivotal role on Glut-4 expression, translocation, vesicular trafficking, and function and vice versa; lower physical activity (lack of training or sedentary lifestyle) reverses these processes [25, 29]. So, disturbing the physiologic process of insulin action through suppressing IST elements' expression/function is the main pathway by which physical inactivity may induce insulin resistance in peripheral tissues.

### 3.2. Beta Cell Insufficiency

Pancreatic beta cells are responsible for insulin synthesis and release, the main hormone in glucose homeostasis, by controlling absorption, digestion, conversion, and storage of carbohydrates [30]. Beta cell insufficiency is a general term mainly referred to as the structural or functional inability of the pancreatic beta cells to fulfill their metabolic activities and impair insulin release in response to meal [31]. It was confirmed that beta cell failure leads to chronic hyperglycemia, which characterizes type 2 DM [31]. Also, inherited abnormalities in beta cell mass or function are important precursors for dysglycemia and type 2 DM [31]. Hence, efficient pancreatic islets are crucial for maintaining normal glucose homeostasis [30, 32, 33]. Furthermore, different levels of beta cell insufficiency are commonly seen in patients with T1DM or T2DM, which have lower levels of circulatory insulin [32, 34, 35]. Therefore, any pathologic factor inducing islet cell dysfunction may be a potential threat to glucose homeostasis [30].

We have strong evidence indicating physical inactivity is closely associated with islet cell insufficiency [36]. An inactive lifestyle can give rise to insulin resistance by increasing islets' workload and lowering their efficiency through various pathways such as ER (endoplasmic reticulum) stress, mitochondrial dysfunction, oxidative stress, and inflammation and promoting the apoptosis and death of beta cells [36]. In contrast, physical training and exercise preserve islets' function and restore it, leading to increased peripheral insulin sensitivity [37]. In addition, they can induce beta cell proliferation via elevations in circulating levels of different growth factors such as growth hormone, IGF-1 (insulin-like growth factor 1), and GLP-1 (glucagon-like peptide 1) [38]. It can also prevent or suppress islet apoptosis and thereby increase the functional mass of beta cells [39].

Slentz and coworkers in 2009 reported that inactive subjects have lower beta cell sufficiency than trained individuals [40]. They found that 8 months of inactivity led to a significant rise in fasting plasma glucose, while moderate- to high-intensity exercise restored these changes, improved islet function, and adjusted glucose metabolism [40]. Also, Dela et al. in 2004 revealed that inactive persons have lesser insulin sensitivity and lower beta cell sufficiency in response to an oral bolus of carbohydrates [37]. Similarly, Lee and coworkers in 2015 found that T2DM patients with lower physical activity had reduced islet function and irregular glucose metabolism compared with the aerobic exercise group [41]. Bloem and Chang, in another trial, reported similar findings indicating even short-term exercise improved pancreatic beta cell activity and glucose metabolism than sedentary individuals [42]. Gomes et al. in 2013 demonstrated that inactive obese diabetic rats have lower pancreatic beta cell function compared with the exercise group [43]. Delghingaro-Augusto and colleagues in 2012 found similar results demonstrating diabetic rats with lower activity have more susceptibility to beta cell failure while exercise improves their function [44]. These studies strongly highlight the strong relationships between physical inactivity and beta cell failure and improvement of islet function with exercise.

### 3.3. Obesity and Dyslipidemia

Obesity, which is commonly associated with dyslipidemia, is closely related to insulin resistance [45]. It has now been accepted as a significant risk factor for DM since it has detrimental effects on different phases of IST, disrupting physiologic insulin signaling pathways toward impaired peripheral insulin sensitivity [45, 46]. Evidence shows that obesity can induce insulin resistance via ER stress induction, oxidative damage, mitochondrial dysfunction, beta cell dysfunction, dysregulation of adipokines and adiponectins involved in glucose homeostasis, impairing expression/localization/activities of IST elements, and evoking and promoting inflammatory processes [45]. Thus, effective preventive or therapeutic approaches against DM are commonly accompanied by lifestyle modification, keeping body weight in a healthy range and physical fitness [4, 47–49].

Physical inactivity and sedentary behaviors can induce insulin resistance via lowering energy expenditure, dysregulating lipid homeostasis, and enhancing lipid storage [46, 50]. Amati et al. in 2009 demonstrated that physical inactivity-dependent obesity underlies insulin resistance in older athletes [51]. Hamburg and coworkers in 2007 found that only five days of bed rest dysregulates serum lipid profile and induces insulin resistance in healthy volunteers [52]. Also, Davies and coworkers in 2018 conducted a clinical study demonstrating a short-term decrease in physical activity dysregulates lipid profile, changes body composition, and increases lipid content and reduces sensitivity inactive participants [53]. They concluded that insulin sensitivity could be improved by normalizing lipid homeostasis and energy balance [53]. Sjöros and colleagues in 2020 reported that physical activity improves cardiometabolic health and lipid profile toward higher levels of insulin sensitivity in sedentary volunteers [54]. In another study, more physical activity was related to lower BMI (body mass index) and lipid content and higher insulin sensitivity in healthy volunteers [55]. Similarly, abdominal obesity and dysregulated lipid profile were reported as underlying culprits of insulin resistance in physically inactive individuals [56]. It must be noted that active people with endurance training may have higher intramuscular lipid, which has no adverse effects on insulin sensitivity (known as athletes' paradox) [57]. This exception may be due to lower DAG or ceramide levels in trained individuals [57]. In total, obesity and dysregulated lipid profiles are potential links between physical inactivity and insulin resistance.

### 3.4. Mitochondrial Dysfunction

Mitochondria are a double-membrane intracellular organelle involved in most metabolic pathways and are recognized as the powerhouse of the cells [58]. It has major roles in vital cellular events such as cell death and signaling, thus playing a fundamental role in body homeostasis [58]. Effective mitochondrial volume is vital for insulin signaling and glucose homeostasis, and any impairment in this pathway increases the risk of insulin resistance [24, 59]. Many patients with diabetes have different levels of mitochondrial dysfunction [60, 61]. It is involved in insulin resistance via at least four mechanisms: point mutations in mtDNA (mitochondrial DNA), activation of PKC (protein kinase C), mitochondria-induced oxidative stress, and pancreatic beta cell dysfunction [59]. Altered mitochondrial capacity or reduced mitochondrial genes involved in glucose homeostasis, such as hexokinase II or PPARGC1A (peroxisome proliferator-activated receptor- *γ* coactivator-1 *α*), were also reported in many patients with T2DM [19, 62]. Moreover, lower mitochondrial content in skeletal muscles was reported in many patients with T2DM [63, 64]. So, any pathologic factor reducing mitochondrial content or function may be a potent risk factor for insulin resistance [59, 65, 66].

There is strong evidence implying physical inactivity and sedentary behaviors have deleterious impacts on mitochondrial function [67, 68]. These findings show that trained individuals have a higher capacity for mitochondrial performances than sedentary individuals [58]. Abadi and coworkers in 2009 reported that limb immobilization significantly downregulates some mitochondrial proteins like cytochrome c oxidase and citrate synthase and suppresses metabolic machinery of glucose homeostasis [69]. Figueiredo et al. in 2009 demonstrated that long periods of inactivity have a deleterious impact on the mitochondrial respiratory function of skeletal muscles in mice [68]. They observed that lifelong inactivity seriously impairs mitochondrial oxidative capacity by inducing oxidative damages in immobilized tissues [68]. Distefano et al. in 2018 found that inactive subjects have lower mitochondrial oxidative capacity than the exercise group [67]. They suggested that active older adults have a better mitochondrial capacity and concluded that mitochondria are a key therapeutic target for sedentary-related complications and insulin resistance [67]. Alibegovic and colleagues in 2010 provided clinical evidence indicating physical inactivity-dependent insulin resistance is closely associated with significant changes in mitochondrial genes involved in glucose metabolism [19]. They found that only 9 days of bed rest impairs PPARGC1A and CPT1B (carnitine palmitoyltransferase 1B) mitochondrial gene expression via downregulation or increased DNA methylation in young men's skeletal muscles [19]. Bilet and coworkers in 2020 provided further evidence implying limb immobilization promotes insulin resistance via suppressing mitochondrial oxidative capacity in skeletal muscles of healthy young men [70]. Therefore, mitochondrial dysfunction is another possible link between sedentary behaviors and insulin resistance and could be an effective therapeutic target for physical inactivity-induced diabetes.

### 3.5. Oxidative Stress

Oxidative stress, which refers to an imbalance between free radicals and antioxidants, favoring the free radicals, is a key player in the pathophysiology of insulin resistance [24, 71]. It can significantly disturb normal IST and disrupt physiologic, metabolic pathways toward pathologic events such as a polyol or hexosamine pathways producing harmful byproducts like AGEs (advanced glycation end products) and MDA (malondialdehyde) [24]. In addition, there is strong evidence suggesting higher levels of free radical species directly attack different elements of IST and disrupt their function and reduce insulin sensitivity [71, 72]. Also, many patients with diabetes have different levels of oxidative stress due to weakened intrinsic antioxidant defenses or hyperproduction of free radicals [73]. Hence, antioxidant therapy in these patients could readjust oxidative balance, improve insulin sensitivity, and normalize whole-body metabolism [74, 75].

We have evidence suggesting that physical inactivity increases oxidative stress [70, 76]. For example, Laufs et al. in 2005 demonstrated that physical inactivity upregulates nox1, p47phox, and p67phox subunits of NADPH oxidase (nicotinamide adenine dinucleotide phosphate oxidase), increases ROS (reactive oxygen species) generation, and induces oxidative damage in vascular tissue of C57BL6 mice [76]. Also, Alghadir and coworkers in 2016 reported that physically inactive patients, compared with active participants, have higher levels of oxidative damage markers as higher MDA and lower TAC (total antioxidant capacity) in plasma [77]. Moreover, Kozakiewicz et al. in 2019 established that inactive older men have lower SOD (superoxide dismutase), CAT (catalase), and GPx (glutathione peroxidase) activity and higher plasma MDA content than active individuals [78]. Accordingly, Alibegovic and colleagues in 2010 reported that physical inactivity insulin resistance is partly dependent on transcriptional changes inducing oxidative stress such as PPARGC1A and TXNIP (thioredoxin-interacting protein), in which daily physical activity reverses these changes [19]. These findings imply that physical inactivity may be correlated to more oxidative damages, disturbing peripheral insulin sensitivity. However, more investigations are required to confirm these findings.

### 3.6. Low-Grade Inflammation

Inflammation is closely involved in the pathophysiology of insulin resistance [79]. Different forms of cytokines and proinflammatory mediators, e.g., TNF-*α* (tumor necrosis factor alpha), MCP-1 (monocyte chemotactic protein-1), and CRP (C-reactive protein), were upregulated in patients with T2DM [79]. Also, animals lacking the proinflammatory mediators were protected against insulin resistance [80]. TNF-*α*, a widely expressed inflammatory cytokine, impairs insulin signaling via serine phosphorylation of IRS-1 or reduces Glut-4 expression [81]. Moreover, other inflammatory pathways such as IKK*β* (a subunit of I*κ*B kinase) and activation of IKK*β*/NF-*κ*b and JNK (c-Jun N-terminal kinase), which is a key element in tissue inflammation, are commonly followed by insulin resistance [82]. Activation of the JNK pathway induces serine phosphorylation in IRS-1 in 307, which impairs insulin signaling (47). Also, another potent cytokine of IL-1 (interleukin-1) reduces IRS-1 expression via ERK1/2 (extracellular signal-regulated kinase 1) and IKK*β*/NF-*κ*b activation in adipocytes and skeletal muscles (48). Likewise, IL-6 stimulates IRS degradation and so reduces insulin sensitivity [83]. Inflammation can also upregulate Socs1 (suppressor of cytokine signaling) and Socs3, which induce IRS degradation through ubiquitylation [84]. Many patients with T2DM have chronic low-grade inflammation with increased accumulation of immune cells and higher levels of circulating proinflammatory markers impairing normal insulin signaling [24, 85]. Thus, any agent that can elicit inflammatory responses, such as inactivity, may threaten insulin sensitivity and glucose homeostasis [79].

Inactivity increases visceral fat accumulation, stimulating chronic low-grade systemic inflammation and dependent comorbidities such as insulin resistance and DM [86]. Thus, there is a vicious mutual cycle between physical inactivity, obesity, and light systemic inflammation, which drives the internal milieu toward insulin resistance [86]. As we know, adipose tissue has endocrine activities by producing and releasing a wide variety of inflammatory factors such as leptin, NY (neuropeptide Y), interleukins, TNF-*α* (tumor necrosis factor alpha), resistin, adipokines, and adiponectin [87]. These proteins have complicated cross-talks with immune system elements and modulate their activity [87]. As a result, lower physical activity is commonly followed by more visceral/subcutaneous adipose tissue (higher BMI), resulting in more immune system activity and higher circulatory levels of inflammatory cytokines [87]. Thus, adipose tissue-induced inflammation is a known cause of insulin resistance in obese people [88, 89].

Studies are suggesting a sedentary lifestyle increases inflammatory markers [86, 90]. In an extensive clinical experiment, Hamer and coworkers found that physical inactivity is directly linked to more circulating inflammatory cytokines [91]. They reported that physical activity has a linear relationship with circulating cytokine levels as CRP (C-reactive proteins) and IL-6 (interleukin 6) in healthy volunteers [91]. Also, Phillips et al., in 2017, conducted a clinical study showing sedentary behavior is associated with higher inflammatory cytokines in plasma [92]. They also found that replacing sedentary behaviors with physical activity reduces circulating cytokines and improves insulin sensitivity in obese adults [92]. Højbjerre et al. in 2011 presented further evidence indicating even short periods of physical inactivity in healthy volunteers can induce inflammatory responses and increase the risk of insulin resistance and T2DM [93]. So, it seems that physical inactivity has a potent relationship with insulin resistance via inducing and promoting inflammatory responses.

### 3.7. Sex Steroids

Sex hormones have dominant impacts on metabolic pathways [94]. These steroids have potent catabolic, anabolic, or releasing effects on main substrates like lipids, proteins, and carbohydrates and could induce or suppress their metabolism in different conditions [94, 95]. Evidence is well confirmed that estrogen (estradiol), progesterone, and testosterone, as the primary sex steroids, have profound effects on most steps of glucose homeostasis such as absorption, glycogenesis, and gluconeogenesis, releasing into circulation and entering into the insulin-dependent cells and thereby providing protective defense against metabolic disorders as well as DM [95]. Testosterone increases Glut-4 expression/localization in adipocytes which in turn increases insulin sensitivity [96]. It also induces insulin sensitivity via IRS phosphorylation [97]. Similarly, estradiol enhances Glut-4 translocation and induces PI3K/Akt signaling pathway, increasing insulin sensitivity [98, 99]. Lower levels of these steroids are closely associated with insulin resistance [100]. For example, age-related or obesity-dependent insufficiency of sex steroids is the main cause of insulin resistance and the onset of T2DM [95]. So, the level of sex steroids and their release have significant importance in glucose homeostasis.

A sedentary lifestyle may alter circulating sex steroids via several pathways as some adipokines (such as leptin and adiponectin) induce estrogen biosynthesis, reducing adiposity, aromatization of androgens (occurs within peripheral adipocytes), and hepatic synthesis of SHBG [101–103]. There is strong clinical evidence confirming these findings. He and colleagues in 2018 found that plasma-reduced sex steroids in plasma are related to higher adiposity and lower physical activity in women and men [104]. They reported that 20 weeks of aerobic exercise significantly increased sex hormones and sex hormone-binding globulin (SHBG) and reduced abdominal fat [104]. Also, Tin and coworkers in 2020 demonstrated that physical activity is directly correlated to levels of sex steroids as estradiol, testosterone, and SHBG in women [105]. Furthermore, they found that self-reported sedentary time is negatively related to plasma levels of these factors [105]. Thus, we have no direct evidence confirming sedentary behavior induces insulin resistance via sex steroids but have indirect evidence. However, more experiments are required to confirm these findings.

### 3.8. Capillarization

Capillarization, which refers to the formation of a network of capillaries in an organ or tissue, is an on-demand process directly associated with the level of metabolism rate in the tissue [106]. Increased capillary density in skeletal muscles is an independent factor predicting the level of insulin sensitivity [107]. Animals with higher capillary density demonstrated a higher glucose tolerance and improved glucose metabolism [106]. Treatment with angiogenic agents improves insulin sensitivity and increases glucose tolerance in animals [108]. Also, tissue-specific insulin sensitivity is directly affected by the level of capillarization in that tissue [106]. Similarly, athletes with higher capillary density and increased blood flow in skeletal muscles have better glucose homeostasis than nonathlete individuals [109], although other molecular mechanisms may also be involved [4]. Although the exact involved molecular pathways are not clear so yet, muscle morphology, the level of angiogenesis, and amount of capillary density are independent determinant factors in insulin sensitivity [110].

It has been confirmed that angiogenesis and capillarization in skeletal muscles are influenced by many factors and metabolites released during physical activity and exercise [111]. Therefore, while exercise and training increase capillarization, physical inactivity and sedentary behavior reduce or suppress this process [106]. Furthermore, studies have shown that an increased level of capillarization is related to more insulin sensitivity, especially in skeletal muscles [106, 112, 113]. For example, Snijders et al., in 2017, conducted a clinical study showing capillary density is a determinant factor for insulin sensitivity in skeletal muscles [110]. They found that muscles with a higher capillary network have better glucose tolerance in response to Oral Glucose Tolerance Test (OGTT) [110]. Also, Rodrigues et al. in 2020 reported that GLP-1 (glucagon-like peptide 1) exerts its antidiabetic effects at least partly via an increase of capillarization in adipocytes [112]. In addition, they found that GLP-1-dependent increased vascular network is correlated to more insulin sensitivity in visceral adipose tissues of rats with T2DM [112]. Moreover, Evans and coworkers recently demonstrated that exercise training and physical activity upregulated angiogenic factors such as VEGF (vascular endothelial growth factor), PlGF (placental growth factor), sFlt-1 (soluble fms-like tyrosine kinase receptor-1), and bFGF (basic fibroblast growth factor) and increased capillarization which accompany improvement in insulin sensitivity in skeletal muscles of older men [114]. These findings suggest that a lower level of capillarization may be another link between sedentary behavior and insulin resistance [115].

### 3.9. Ceramide Level

Ceramide is a family of naturally occurring highly bioactive lipids present abundantly in the lipid bilayer membranes of eukaryotic cells and contribute to many intracellular pathways, such as free radical generation, the release of inflammatory cytokines, apoptotic processes, and gene expression [116]. These lipid molecules are mainly composed of sphingosine and are a significant component of the cellular lipid bilayer membrane by an essential role in maintaining its integrity [117]. Ceramide synthesis occurs in at least three distinct ways as the de novo pathway, the sphingomyelin hydrolysis (degradation), and the salvage (recycling) pathway [8]. In addition to structural roles, more recent studies have suggested a causal relationship between ceramide and metabolic complications as well as insulin resistance [118]. They have shown that ceramide may play a role in pancreatic inflammation, beta cell apoptosis and insulin synthesis, ER stress, adipokine release, mitochondrial stress, IRS-1 phosphorylation, and oxidative stress [119, 120]. Furthermore, treatment with myriocin, an inhibitor of de novo ceramide synthesis, has improved insulin sensitivity [121]. Also, the knockout of ceramide-generating enzymes in animals has increased insulin sensitivity [122, 123]. Therefore, ceramide is now widely accepted as a potent insulin antagonist involved in the pathophysiology of insulin resistance and DM, especially in overweight and obese people [116].

There is strong evidence suggesting physical inactivity increases ceramide production, which induces insulin resistance [124]. Bergouignan and colleagues in 2009 have reported that physical inactivity declines insulin sensitivity via impairing cellular and plasma trafficking and metabolism of lipids as well as ceramides in lean women [125]. They observed that 2 months of bed rest was followed by saturated fat and sphingosine accumulation in myocytes, which impaired insulin sensitivity in participants [125]. Also, Kwon and coworkers in 2015 established that 14 days of inactivity increases ceramide level, dysregulates skeletal muscle insulin signaling, and impairs glucose tolerance in mice [126]. Furthermore, Bergman et al. in 2016 provided direct evidence indicating the increased amount of muscle ceramide during physical inactivity is related to insulin resistance in obese volunteers [127]. They found that acute exercise reduces sphingolipid synthesis in the recovery period and improves insulin sensitivity in trained volunteers [127]. However, due to some reports about the effect of short-term inactivity on ceramide level and glucose homeostasis, it seems that ceramides need more time to exert pathologic effects and disturb insulin signaling [128].

## 4. Conclusion

Physical inactivity, a common health risk of the modern lifestyle, is a severe threat to body homeostasis that deviates physiologic metabolism toward injurious pathways. So it is now recognized as a potent underlying cause of insulin resistance and DM, but the interconnections are not fully understood. Our study suggests that physical inactivity is closely related to insulin resistance via at least 9 molecular mechanisms (Table 1) as genetic modulation of IST elements, impairment of pancreatic beta cell function, increase of the risk of dyslipidemia and obesity, mitochondrial dysfunction, increase of oxidative damages, modulating sex hormone expression/function, reduction of the vascular network as capillarization, enhancement of ceramide production, and inducing chronic low-grade systemic inflammation. We have strong clinical evidence regarding the links with these various pathways; however, other unidentified cellular pathways may contribute to this.

## Figures and Tables

**Table 1:** Cellular pathways linking physical inactivity and insulin resistance (IST = insulin signal transduction).

Molecular mechanisms	Effects of physical inactivity	Experimental evidence	Clinical evidence
Genetic modulations	Modulates expression/function of IST elements	[26]	[18, 19]
Beta cells' insufficiency	Induces beta cell insufficiency and reduces pancreatic islet mass	[43, 44]	[37, 40–42, 129]
Obesity and dyslipidemia	Reduces energy expenditure toward dyslipidemia and higher risk of obesity which in turn stimulates insulin resistance	—	[50–53]
Mitochondrial dysfunction	Reduces mitochondrial mass, which in turn impairs insulin expression/secretion/signaling	[68]	[19, 67, 69, 70]
Oxidative damages	Increases free radical species followed by more systemic oxidative stress	[76]	[77, 78]
Inflammation	Onset and progress low-grade inflammatory response, which in turn induce insulin resistance	—	[91–93]
Sex steroids	Modulates sex steroid expression/secretion leading to impaired glucose homeostasis	—	[104, 105]
Capillarization	Reduces the amount of vascular network, which in turn impairs insulin sensitivity	—	[106, 112]
Ceramide synthesis	Increases the amount of ceramide synthesis, which in turn interferes with insulin signaling	—	[125–127]

## Data Availability

There is no raw data associated with this review article.
